# Scale Invariance Properties of Intracerebral EEG Improve Seizure Prediction in Mesial Temporal Lobe Epilepsy

**DOI:** 10.1371/journal.pone.0121182

**Published:** 2015-04-13

**Authors:** Kais Gadhoumi, Jean Gotman, Jean Marc Lina

**Affiliations:** 1 Montreal Neurological Institute, McGill University, Montréal, Québec, Canada; 2 Département de Génie Électrique, École De Technologie Supérieure, Montréal, Québec, Canada; 3 Centre de recherches mathématiques, Université de Montréal, Montréal, Québec, Canada; Universiteit Gent, BELGIUM

## Abstract

Although treatment for epilepsy is available and effective for nearly 70 percent of patients, many remain in need of new therapeutic approaches. Predicting the impending seizures in these patients could significantly enhance their quality of life if the prediction performance is clinically practical. In this study, we investigate the improvement of the performance of a seizure prediction algorithm in 17 patients with mesial temporal lobe epilepsy by means of a novel measure. Scale-free dynamics of the intracerebral EEG are quantified through robust estimates of the scaling exponents—the first cumulants—derived from a wavelet leader and bootstrap based multifractal analysis. The cumulants are investigated for the discriminability between preictal and interictal epochs. The performance of our recently published patient-specific seizure prediction algorithm is then out-of-sample tested on long-lasting data using combinations of cumulants and state similarity measures previously introduced. By using the first cumulant in combination with state similarity measures, up to 13 of 17 patients had seizures predicted above chance with clinically practical levels of sensitivity (80.5%) and specificity (25.1% of total time under warning) for prediction horizons above 25 min. These results indicate that the scale-free dynamics of the preictal state are different from those of the interictal state. Quantifiers of these dynamics may carry a predictive power that can be used to improve seizure prediction performance.

## Introduction

The existence of a preictal state distinguishable from an interictal state of the epileptic brain is supported by growing evidence of electrophysiological changes preceding the ictal phase [1–6]. Transitions from interictal to ictal state are likely to be governed by various mechanisms [7–9] with different electrophysiological manifestations. Revealing changes in dynamical properties of the electroencephalogram (EEG) related to state transition is probably a multifaceted problem and solving it requires a combination of tools each adapted to reveal changes in a distinct aspect of signal properties. For example, preictal changes are probably better detected using a combination of intra- and inter-channel EEG information rather than using either information alone [10].

Many studies in seizure prediction adopted the approach of combining EEG features that capture different properties of the signal [11–16]. Whether reported prediction performances are superior to those achieved with single feature algorithms is yet to be verified. It remains intuitive though that an increase in the prediction performance is expected when combining the predictive power of different methods in a complementary manner.

In this study, we examine whether combining measures of two a priori unrelated properties of the EEG, the so-called thermodynamic and scale invariance properties, would result in an improvement of the prediction performance. Descriptors of the thermodynamic property of the intracerebral EEG, namely the wavelet energy and entropy, have been introduced in our previous study [1] and estimated in the high frequency range (50–450 Hz). We demonstrated that measures derived from these descriptors were useful to achieve significant and practical seizure prediction performance results [17]. Scale invariance is a property related to temporal irregularity in signals, first introduced in the study of turbulence [18]. Evidence of scale invariance in the intracranial EEG and its significance to brain electrophysiological function has been demonstrated in [19]. Here, we analyze the scale invariance property of the intracerebral EEG via descriptors extracted through a novel scaling analysis framework: the bootstrap and wavelet leader based multifractal analysis [20,21].

Using scaling analysis based descriptors, we investigate the discriminability of preictal and interictal EEG epochs in a group of patients with intractable mesial temporal lobe epilepsy. We then assess the prediction performance of our previously proposed patient-specific classifier using these descriptors individually and in combination with our previously introduced state similarity measures.

## Materials and Methods

### Ethics statement

This study was approved by the Montreal Neurological Institute and Hospital Research Ethics Board (REB). The REB acts in conformity with standards set forth by the Tri-Council Policy Statement and Ethical Conduct for Research Involving Humans (Canada), and by the Rules and Regulations of the Department of Health and Human Services and the Food and Drug Administration (US) governing human subjects research and functioning in a manner consistent with internationally accepted principles of good clinical practice. All adult subjects signed an REB-approved written informed consent form.

### Materials

To ensure an unbiased comparison between the performance of scaling analysis based features and originally proposed state similarity measures, we use the same dataset as in our previous work [17]. Long-lasting intracerebral EEG (iEEG) recordings, low-pass filtered at 500 Hz and sampled at 2000 Hz, from 17 consecutive patients who attended the Montreal Neurological Institute, Montreal, Canada, between 2004 and 2011 and who underwent depth electrode investigation of refractory epilepsy, were analyzed. The selected patients had a mesial temporal lobe epilepsy (MTLE) diagnosis. They had at least five seizures recorded at 2000 Hz during the investigation.

Depending on the number of implanted electrodes, nine to 18 bipolar iEEG channels from the four deepest contacts of depth electrodes targeting the bilateral mesial temporal structures (amygdala, hippocampus and parahippocampus) were selected. A total of 1565 hours and 175 seizures were analyzed. Electrographic onsets of seizures were determined by an experienced neurologist. Only seizures that are at least two hours apart were considered in the analysis in order to minimize the postictal effect.

The iEEG data was split into training and testing subsets for each patient. The training subset has two or three preictal epochs of uninterrupted iEEG recordings lasting between six and 22 min across patients, and five interictal epochs lasting almost one hour each and separated by at least one hour from each other and by four hours from any seizure. The training data were selected from the beginning of the patient’s iEEG recordings. The remaining iEEG data were used for testing. They are made of continuous multiday iEEG recordings. The number of test seizures per patient ranged between three and 24. In total, 119 hours of iEEG data were used in training and 1446 hours in testing (see S1 Table for a list of training and testing data duration and number of test seizures for each patient). Patient clinical characteristics, surgery outcomes and iEEG data are summarized in Table 1.

**Table 1:** Summary of iEEG dataset and seizure onset.

**Patient**	**Sex/Age**	**Seizure laterality**	**Channels analyzed**	**# channels analyzed**	**Surgery outcome** * **/ follow-up period**	**# of seizures recorded**	**# of seizures analyzed**	**# of iEEG hours analyzed**
1	M/29	Bil., R>L	A, H, RP	15	I (2.5y)	8	7	56.8
2	F/42	R	A, H, P	18	II (3y)	9	9	132.5
3	F/44	Bil., L>R	A, H, P	18	III (3.5y)	6	6	47.7
4	M/46	R	A, H, P	18	IV (3y)	9	8	110.7
5	F/40	L	A, H, P	18	I (2y)	30	10	57.7
6	F/53	Bil., L>R	A, H, P	18	n/a	7	6	84.4
7	M/24	R	RA, RH, RP	9	II (3y)	8	8	109.6
8	M/25	Bil, R>L	A, H, RP	15	IV (2y)	6	7	147.7
9	M/44	L	A, H, P	18	II (1y)	6	6	140.8
10	F/30	Bil., R>L	A, H, P	18	IV (2y)	18	17	52.8
11	F/47	Bil., L>R	A, H, P	18	n/a	31	23	55.6
12	M/28	Bil., R>L	A, H, P	18	III (3y)	27	27	39.6
13	M/23	Unclear	A, H	12	n/a	9	9	17.4
14	M/38	R	A, H	12	III (3y)	6	6	89
15	M/33	Bil.	A, H, P	18	n/a	7	17	169.5
16	M/21	R	RA, RH, RP	9	n/a	13	13	143.1
17	F/28	Bil., R>L	A, H, P	18	IV (1y)	6	6	110.4

* Based on the International League Against Epilepsy post-surgical outcome classification.

R: Right. L: Left, A: Amygdala, H: Hippocampus, P: Parahippocampus.

Bil.: Bilateral, >/< designate preponderance (based on 70% or more of number of seizures originating from one side).

y: year, m: month.

### Theoretical background of scaling analysis

Scale invariance or “*scaling*” is defined as the absence of a particular time scale playing a characteristic role in the process [22]. Such a process is called a “*scale free*” process. For stochastic processes such as in the case of EEG, scale invariance implies that the statistical properties at different time scales (e.g., hours versus minutes versus seconds) effectively remain the same [23]. Implicitly, scaling analysis identifies and characterizes the rules describing the relation between different scales. These rules are principally governed by power laws. Practical scaling analysis aims mostly at estimating the exponents that characterize these power laws: If *X* designates an EEG signal from one channel and *T*_*X*_(*a*, *t*) a multi-resolution measure of the content of *X* around a time *t* and a scale *a*, then the scale invariance property of *X* is described by the power law behaviors of the time average of the *q* ^th^ power of *T*_*X*_(*a*, *t*) with respect to the analysis scale *a* for a given (large) range of scales *a* ∈ [*a*_*m*_, *a*_*M*_], *a*_*M*_/*a*_*m*_ >>1.

$$\frac{1}{n_{a}}\overset{n_{a}}{\underset{k=1}{\sum }}{\left|T_{X}\left(a,k\right)\right|}^{q}∼a^{\zeta \left(q\right)}$$ (1)

with *n*_*a*_ the number of samples of *X* at the scale *a*. For a range of order *q* values, the set of estimated exponents *ζ*(*q*) fully describes the statistical distribution of the signal and provides a powerful means of characterizing the scale invariance of the signal.

### Models of scaling analysis

Various processes exhibiting scale invariance properties are used as models for scaling analysis. The following summarizes some of the most studied models.

- Self-similarity: the framework of “*self-similarity*” [24] is one of the first models that fulfills the scale invariance property in a mathematically simple and precise way [25]. A random process *X(t*) is said to be self-similar if:

$$X\left(t\right)\overset{d}{=}a^{H}\;X\left(\frac{t}{a}\right),\;\forall a>0,$$ (2)

Where $\overset{d}{=}$ designates equality of statistical properties. The parameter *H*, called *Hurst exponent*, defines and controls the scale invariance of the process *X(t)*.

- 1/f processes: for many real-data processes, scale invariance may exist only for a range of scales and/or the scaling exponents cannot be described with a single parameter. For such processes, self-similarity is not a suitable model. The so-called 1/*f* processes are more flexible models of scale invariance that address the limitations of the self-similarity model. These are processes for which the spectral density obeys a power law with a sufficiently large range of frequencies:

$$S_{X}\left(f\right)∼\frac{1}{f^{\alpha }}\;,\;\;\;\;\;\;f_{m}<f<f_{M}$$ (3)

where *α* is a scalar referred to as the “index of long-range dependence”. It is related to the Hurst exponent by:

α=2H−1 (4)

**Fig 1:**
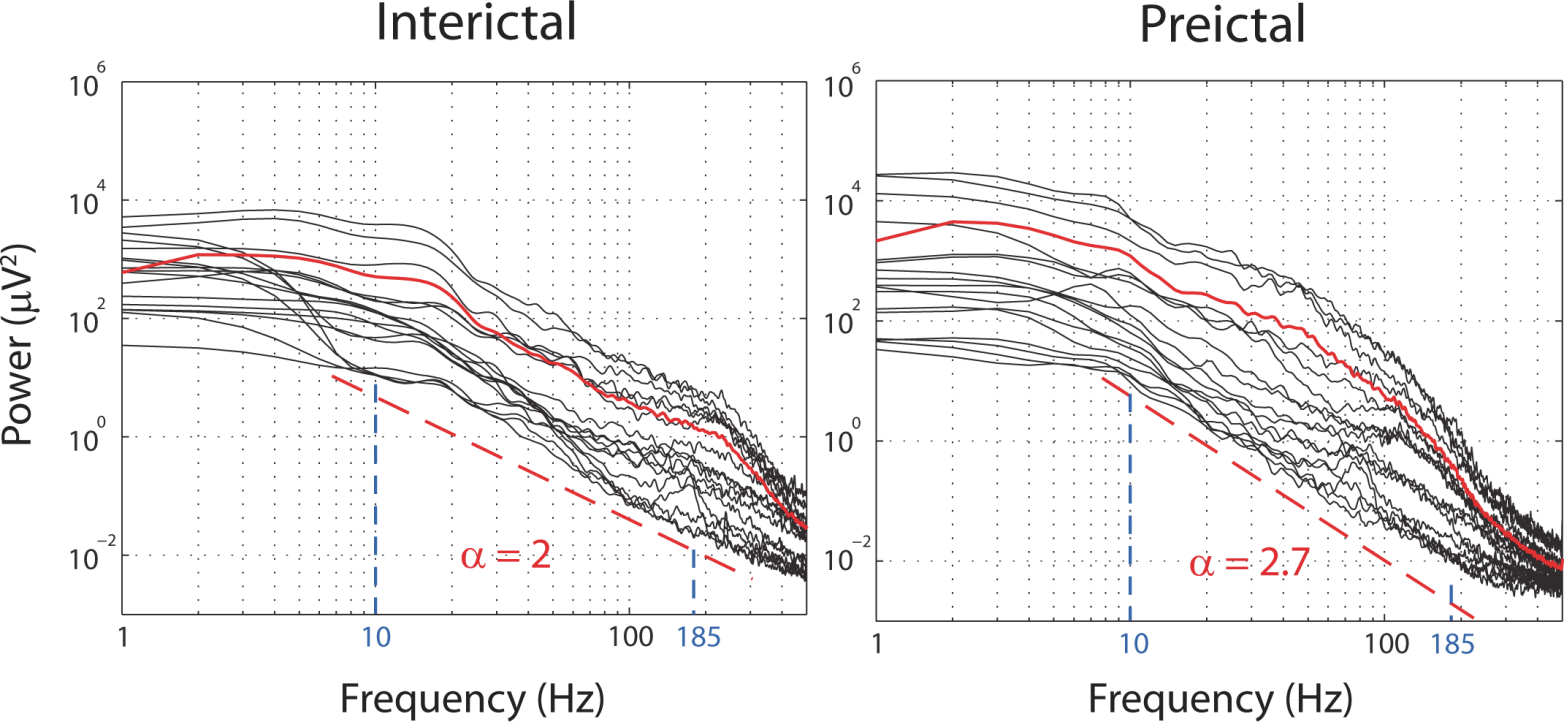
Power spectra of 1-min interictal (left) and preictal (right) epochs from 18 iEEG channels (patient 15). Power spectrum in red is the average power spectrum of all channels. Both epochs show a power law behavior (*P*(*f*) ∼ 1/*f* ^*α*^) illustrated with the fitted dotted red line for the range of frequencies delimited with a dashed blue line (corresponding to the range of scales *j* between 3 and 7). The exponents *α* obtained by a least-square fit are different in the preictal and interictal epochs.

Fig 1 illustrates a preictal and an interictal iEEG epochs exhibiting a 1/f process. Two particular 1/*f* processes define two interesting models, namely long-range dependency [26] and monofractality.

- Long-range-dependency (LRD): LRD is a model defined for scaling observed in the limit of small frequencies (equivalently, large scales), *f*_*m*_ → 0 in Eq (3). It is usually defined in terms of covariance properties relevant to second-order stationary processes. In long-range dependent processes, the temporal correlation between values decays very slowly, allowing for a dependency between past and future values.

- Fractals and multifractals: fractal processes are models of scale invariance described through the local (*Hölder*) regularity of the process sample path measured by the Hölder exponents *h(t)*:

$$\left|X\left(t+\lambda \right)-X\left(t\right)\right|\approx c\lambda^{h\left(t\right)},\;\;\;\;\;\lambda >0,c\in R^{+}$$ (5)

The local regularity is the degree of smoothness of the signal in a small time interval. Strong local regularity indicates a smooth signal and weak local regularity indicates less smoothness.

When the Hölder exponents *h(t)* are a deterministic and stationary function, the process exhibits a slow and smooth regularity fluctuation over time and is called *fractal*.

**Fig 2:**
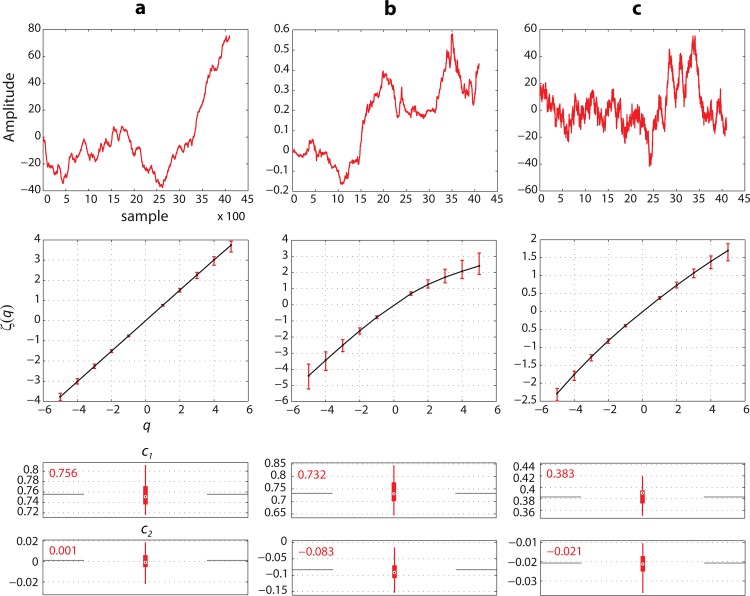
Estimation of cumulants *c*_1_ and *c*_2_ using wavelet leader and bootstrap based scaling analysis in signals with different scale invariance properties. Each signal is 4096 samples. 100 bootstrap wavelet leader resamples were used in cumulant estimation. (a) Realization of a self-similar signal (fractional Brownian motion). ζ(*q*) is linear in *q*. *c*_1_ ≠ 0 and *c*_2_ ≈ 0. (b) Realization of a multifractal random walk. ζ(*q*) is nonlinear in *q*. *c*_1_, *c*_2_ ≠ 0. (c) EEG channel recording showing nonlinear relation of ζ(*q*) in *q*. *c*_1_, *c*_2_ ≠ 0. (Each column, top to bottom: Signal plot, regression plot of ζ(*q*) exponent estimates and boxplot of cumulant estimates).

When the Hölder exponents *h(t)* are the same for all *t*, the process exhibits constant regularity along its sample paths; it is referred to as *monofractal* process. An example of monofractal process in the fractional Brownian motion (Fig 2.A).

When the fluctuation of *h(t)* is random and highly undeterministic, the process is said to be *multifractal*. Instead of Hölder exponents, the irregularity of such processes is described rather through a spectrum *D(h)* called the singularity (multifractal) spectrum [27,28]. An example of a multifractal process is the multifractal random walk (Fig 2.B).

### Wavelet leader based scaling analysis

Wendt et al. [21] proposed a scaling analysis framework for a robust estimation of the scaling exponents using *cumulants* as surrogate measures of the exponents. In the proposed framework, wavelet leaders [29]—quantities derived from the discrete wavelet analysis of the signal—are used as a multi-resolution measure in Eq (1). Using a mathematical formalism (see S1 appendix), it was established that the scaling exponents *ζ*(*q*) can be estimated through the cumulants *c*_*p*_ of the Taylor series polynomial expansion:

$$\zeta \left(q\right)=\overset{\infty }{\underset{p=1}{\sum }}c_{p}\frac{q^{p}}{p!}\;$$ (6)

The advantage of using the cumulants as surrogate measures of the scaling exponents lies in the fact that they emphasize the departure from linear behavior of *ζ*(*q*) in *q*: there exists *p* ≥ 2: *c*_*p*_ ≠ 0 [30].

From a single path (single observation) of the signal, the cumulants *c*_*p*_ can be estimated through statistical models and bootstrap approaches. At each scale, *R* bootstrap resamples are generated from the wavelet leader coefficients. *R* bootstrap cumulant estimates are calculated and then averaged to obtain the final estimates. In practice, the first few cumulants are sufficient to gather most of the scaling property from the signal [21,31]. For *c*_1_ ≠ 0, knowing whether *c*_2_ ≠ 0 is practically equivalent to choosing between monofractal and multifractal models of scale invariance. In this study, we use the first and second cumulants, *c*_1_ and *c*_2_.

Fig 2 shows examples of cumulant estimates from realizations of simulated signals with different scale invariance properties and from a bipolar recording of an intracranial EEG channel.

### Cumulant estimation

The first two cumulants *c*_1_ and *c*_2_ are calculated using the *Wavelet Leader and Bootstrap based MultiFractal analysis (WLBMF)* toolbox (http://www.irit.fr/~Herwig.Wendt/software.html) which implements the empirical multifractal analysis formalism proposed by Wendt et al. [21]. Cumulants were estimated in the training and testing data for scales *j* between 3 and 7 (corresponding to a frequency range between 11.8 Hz and 187.5 Hz for which the power spectrum exhibits a 1/f-like behavior, see Fig 1) and moments of order *q* between -5 and 5 in 2s consecutive non overlapping sliding windows using 100 bootstrap resamples of wavelet leaders. A *Daubechies* wavelet with 3 vanishing moments (Daubechies 3) was used as a mother wavelet. Then the average cumulant was calculated in a 1-min window sliding at a 15s step. The length and overlap of the latter window were selected to match those used in our previous work [17]. This is to warrant an unbiased evaluation of the cumulants when comparing the performance of the seizure prediction method using cumulants to that using state similarity measures.

### Statistical comparison of preictal and interictal cumulants

In each patient independently, we compared the values of cumulant observations in 5-min preictal and 5-min interictal epochs of the training dataset. We hypothesized that the median of the cumulant observations does not statistically change when sampled from a 5-min preictal or from a 5-min interictal epoch. To verify this hypothesis, we compared average cumulant observations from the 5-min preictal and the 5-min interictal epochs using an unpaired two-tailed Wilcoxon rank-sum statistical test of the equality of medians. Preictal observations from available 5-min preictal training data were averaged. Similarly, interictal observations from consecutive 5-min epochs of training interictal data were averaged. The statistical comparison was carried in each channel independently and a Bonferroni correction was performed to control for multiple (channels) comparisons.

### Cumulant versus spectral power

A change in the state of vigilance (sleep/wakefulness) is generally accompanied by a significant change in the spectral power. This could affect the values of cumulant estimates as they indirectly measure inter-band frequency relations. To control for the state of vigilance as a possible confounder in the preictal and interictal cumulant comparison, we assessed whether any difference between preictal and interictal cumulant observations could only be due to a difference in the state of vigilance. Using the same sliding window we used to compute the average cumulant estimates (i.e. 1-min length, 15s step), we calculated the mean spectral power in the preictal and interictal epochs of the training dataset in the conventional EEG frequency bands: 0.5–3.9 Hz (delta), 4–7.9 Hz (theta), 8–14 Hz (alpha), 15–30 Hz (beta), 30–58 Hz (gamma), 80–250 Hz (ripples) and 250–450 Hz (fast ripples). The average 5-min preictal and interictal spectral power observations were then calculated the same way the average cumulant observations were obtained. By averaging the spectral power across multiple epochs, the confounding effect of the state of vigilance on the comparison between cumulant observations is minimized. The discriminability between preictal and interictal states using cumulants is tested by evaluating the difference between average preictal and interictal cumulant observations and comparing this difference to that observed between average spectral powers.

### Seizure prediction using cumulant features

**Fig 3:**
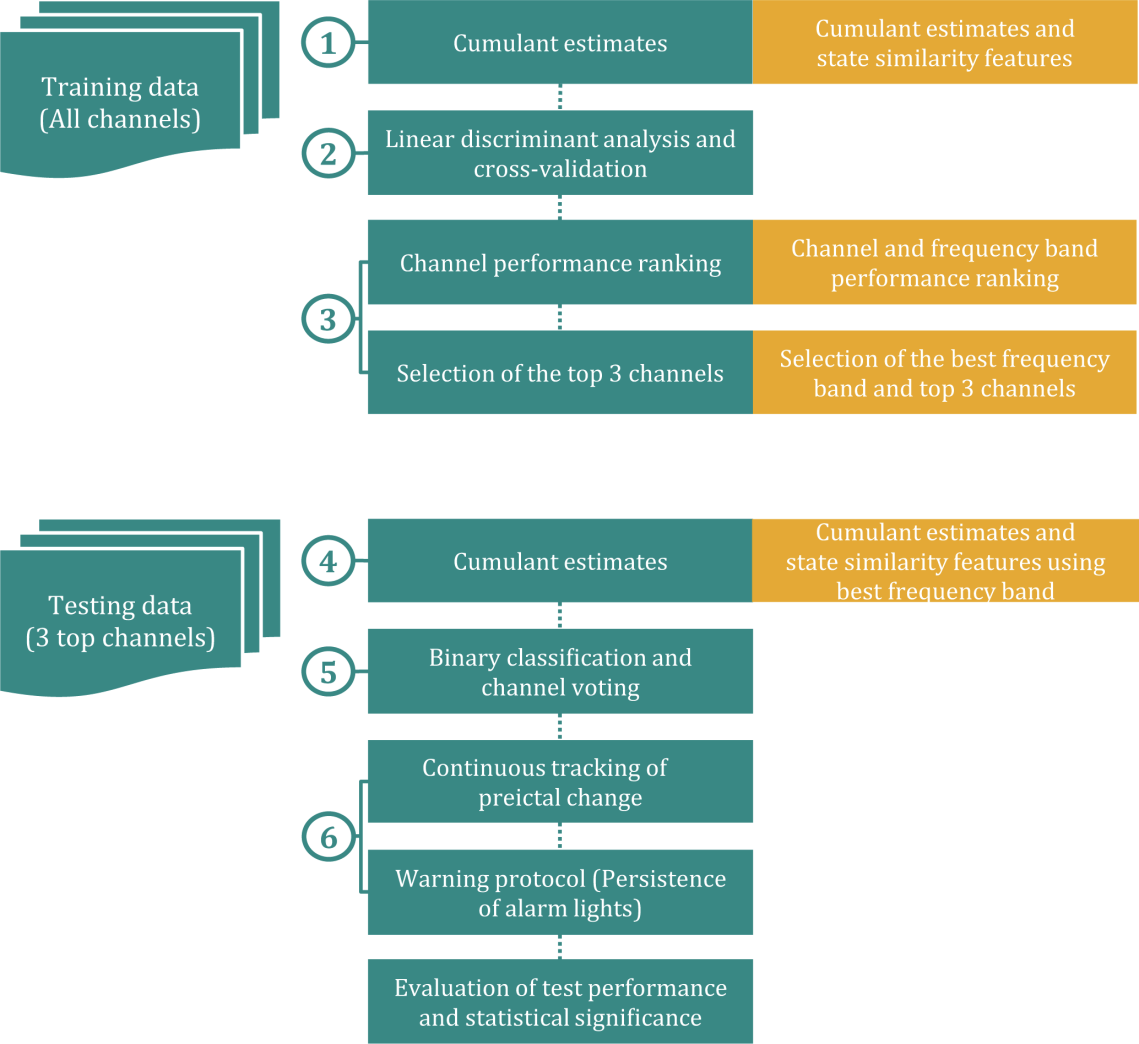
Flow chart of the seizure prediction method. Steps 1 to 3 are training procedures and 4 to 6 are testing procedures. Boxes in orange show procedures used instead when combination of features are used.

Our original patient-specific seizure prediction method [17] uses a set of three measures, namely the *persistence*, *distance* and *inclusion*, referred to as *state similarity measures*, which quantify the similarity between the states underlying iEEG epochs and a reference state underlying the immediate preictal (90s) iEEG epoch. The state similarity measures are derived from the two dimensional thermodynamic profiles of the iEEG epochs obtained from time-series of wavelet energy and entropy calculated using the wavelet transform modulus maxima method (WTMM). We adapted our prediction method to use cumulants individually and in combination with state similarity measures as feature sets to predict seizures, with no change to the parameters used in training and testing procedures of the original algorithm. Fig 3 depicts a flow chart of the training and testing steps of the adapted version of the method. The outline of these steps is presented below. A detailed description of the concepts and procedures involved in each step can be found in the aforementioned reference.

### Training procedures

All training procedures for the optimization of the classifier are performed on training data.

*Step 1- Feature computation*: Cumulant estimates *c*_1_ and *c*_2_ (and state similarity measures) are calculated for each iEEG channel of the training dataset as previously described.
*Step 2- Discriminant analysis*: A 10-fold leave-one-out cross validation is performed across preictal and interictal feature observations. A linear discriminant analysis is performed in each iteration. The classifier performance in each channel is iteratively assessed using a score combining the sensitivity and the specificity of the classification.
*Step 3- Performance assessment*: Final channel scores are ranked and the top three channels with the highest scores are selected for testing procedures. When using state similarity measures, four frequency bands are independently assessed in each channel. In addition to channel selection, the frequency band yielding the best performance is also selected for testing procedures.

### Testing procedures

All testing procedures to assess the classifier are performed on test data in a quasi-prospective manner.

*Step 4- Feature computation*: All features (cumulant estimates *c*_1_ and *c*_2_ and state similarity measures) are calculated for top ranked EEG channels (and selected frequency bands in the case of state similarity measures) in the sliding window.
*Step 5- Classification*: Windows are labeled as ‘*preictal’* or ‘*interictal’* by the classifier in each channel separately. Channel decisions are then aggregated using majority voting rule for an ultimate classification of the window.
*Step 6-*: *Preictal change detection*: Preictal changes are signaled through ‘*preictal’* classification of five consecutive windows, corresponding to 2 min of iEEG continuously classified as ‘*preictal’*. Warnings are then raised for a variable period of time (referred to as period of active warning) controlled with the *persistence-τ* parameter. Raised warnings remain active for as long as a preictal change is detected, according to the persistence of warning lights protocol [32]. The parameter persistence-*τ* is equivalent to sum of the seizure occurrence period (the period during which the seizure is to be expected) and the seizure prediction horizon (the minimum time between the warning and the beginning of the seizure occurrence period) as defined in [33]. Warnings followed by iEEG interruptions lasting above three minutes are discarded since it is unknown if the patient was in ictal or interictal phase during this time and the classifier outcome could not be assessed.

### Statistical validation

The performance of the algorithm is compared to that of a chance predictor to assess the statistical significance of its prediction power. The improvement over chance is evaluated through a statistical comparison between the sensitivity of the algorithm and the sensitivity of the chance predictor which is demonstrated to be equal to the proportion of time spent in warning *ρ* [32]. More explicitly, we calculate the *p*-value of the significance of improvement over chance, given analytically in Equation (7), to evaluate the superiority of the prediction performance to chance at the 5% significance level.

$$p=1-\overset{n-1}{\underset{k=0}{\sum }}\left(\begin{array}{c} N\\ k \end{array}\right)\rho^{k}{\left(1-\rho \right)}^{N-k},\text{ for }\frac{n}{N}\geq \rho$$ (7)

where *n* is the number of seizures correctly predicted and *N* the total number of seizures.

### Comparison of prediction performances

Six combinations of cumulant and state similarity measures (feature sets *FS*1 to *FS*6) were used in the seizure prediction method: *FS*1: *c*_1_, *FS*2: *c*_2_, *FS*3: *c*_1_ combined with *c*_2_; *FS*4: State similarity measures combined with *c*_1_; *FS*5: State similarity measures combined with *c*_2_ and *FS*6: State similarity measures combined with *c*_1_ and *c*_2_. Using the testing dataset, the prediction performance of the algorithm was evaluated for each feature set. The sensitivity, the proportion of time spent under warning, and the warning rate were assessed for ten persistence-*τ* values between five and 60 min. The false positive rate is also assessed for comparability with existing seizure prediction methods which reported the specificity in terms of false positive rate. The improvement over chance is assessed for each persistence-*τ* value separately. The sensitivity is defined as the proportion of test seizures that occurred within the period of active warning. The proportion of time spent under warning is the total time of active warning periods divided by the total time of test data. The warning rate is the number of all warnings divided by the total time of test data. The false positive rate is defined according to the recommendation of Mormann et al. [34]. It is the number of warnings not followed by a seizure occurrence within the period of active warning, divided by the total time of test data excluding the time of assumed preictal periods. The latter is defined as the sum of the period of time preceding each test seizure for which a false prediction cannot occur by definition. The time of assumed preictal periods can be obtained by multiplying the number of test seizures by the value of persistence-*τ*.

### Correction of multiple comparisons

To correct for multiple comparisons between the sensitivity of the chance predictor and that of the proposed prediction method (in total 1224 comparisons corresponding to 17 patients, 12 values of persistence-τ, and 6 feature sets), we controlled the false discovery rate (the expected proportion of false discoveries amongst the rejected hypotheses) by calculating the *q*-values (corrected *p*-values) from the *p*-values obtained by single comparisons, using the *Benjamini-Hochberg* procedure [35]. A *q*-value less 5% indicates that above-chance prediction is possible for the corresponding feature set and value of persistence-τ in the given patient.

## Results

### Comparison between preictal and interictal cumulant and spectral power observations

To find out whether a difference between average preictal and interictal cumulants is not only due to a difference in spectral power, we evaluated the association between the significance of difference in cumulants and that of the difference in the power of each analyzed EEG spectral band. The comparison between the average 5-min preictal and 5-min interictal cumulant observations was performed for all channels at the 1% significance level (Bonferroni corrected for multiple channel comparisons). For each cumulant and for each patient, channels that showed a statistically significant difference between interictal and preictal epochs were ranked according to the *z*-score of the comparison test. The most discriminating (highest *z*-score) channel in each cumulant was then used to compare the spectral power in preictal and interictal epochs in the frequency bands analyzed. Results of the difference in cumulants and in spectral power are detailed in Table 2. For all patients, at least one channel showed a statistically significant difference in *c*_1_ and in *c*_2_. This difference was highly significant (corrected *p*-value < 0.001) in 15 of 17 patients (88%) for both cumulants and less significant (corrected *p*-value < 0.01) in the other 2 patients. The most discriminating channel in *c*_1_ was the same as in *c*_2_ in 10 of 17 patients (59%). In 11 of 17 patients (65%), the average *c*_1_ was higher in the preictal observations than in the interictal observations. For *c*_2_, 8 of 17 (47%) patients showed a higher value of the average *c*_2_ in the preictal observation compared to the interictal observations.

**Table 2:** Significance of the difference in the average cumulant and the spectral power observations between preictal and interictal epochs (5-min length) using the most discriminating channel in *c*_1_ and the most discriminating channel in *c*_2_.

**Patient**	**Cumulants**		** **	**Spectral bands**						
	***c***_1_	***c***_2_	** **	**Delta**	**Theta**	**Alpha**	**Beta**	**Gamma**	**Ripples**	**Fast ripples**
1	++ (↑)			++	++	-	-	++	++	++
		++ (↓)		++	++	-	-	++	++	++
2	+ (↑)			-	-	-	-	-	-	-
		++ (↑)		-	-	-	-	-	-	-
3	++ (↑)			-	-	-	-	-	-	++
		++ (↓)		-	-	-	-	-	-	++
4	++ (↓)			++	++	-	++	+	-	-
		++ (↑)		++	++	-	++	+	-	-
5	++ (↑)			-	-	++	++	-	++	++
		++ (↑)		-	-	++	++	-	++	++
6	++ (↓)			++	++	++	++	-	++	-
		++ (↓)		++	++	++	++	-	++	-
7	++ (↑)			-	-	-	-	+	++	-
		++ (↑)		-	-	-	-	+	++	-
8	++ (↓)			-	-	-	-	-	-	-
		++ (↓)		-	-	-	-	-	-	++
9	++ (↓)			-	-	-	-	-	++	++
		++ (↑)		-	-	-	-	-	++	++
10	++ (↑)			++	-	-	-	++	++	++
		++ (↓)		-	-	-	+	++	++	++
11	++ (↑)			+	-	-	-	-	-	+
		++ (↑)		+	+	-	-	-	-	-
12	++ (↑)			-	-	-	-	-	-	++
		++ (↑)		-	-	-	-	-	-	++
13	++ (↓)			++	++	++	-	-	-	-
		++ (↓)		-	+	++	++	++	-	-
14	++ (↑)			-	-	-	-	-	-	++
		++ (↓)		-	-	-	-	-	-	++
15	++ (↓)			++	-	++	-	-	++	++
		++ (↑)		++	-	++	-	-	++	++
16	+ (↑)			++	-	-	++	++	++	++
		++ (↓)		++	++	-	-	-	+	++
17	++ (↑)			++	++	++	++	++	-	-
		++ (↑)		++	++	++	++	++	-	-
Total*	17			9	5	5	5	6	8	10
		17		7	7	5	6	6	8	10

++ (corrected *p*-value < 0.001), + (corrected *p*-value < 0.01), —(no significant difference)

↑ (mean preictal cumulant > mean interictal cumulant)

↓ (mean preictal cumulant < mean interictal cumulant)

* Total shown is the number of patients in whom a significant difference is observed (corrected *p*-value < 0.01) in the spectral band power. None of the spectral bands showed a statistically significant difference in the spectral power for all patients (i.e. no single spectral band showed a correlation between the difference in cumulants and the difference in spectral power in all patients), suggesting that the observed difference in cumulants is likely not the result of a difference in spectral power.

**Fig 4:**
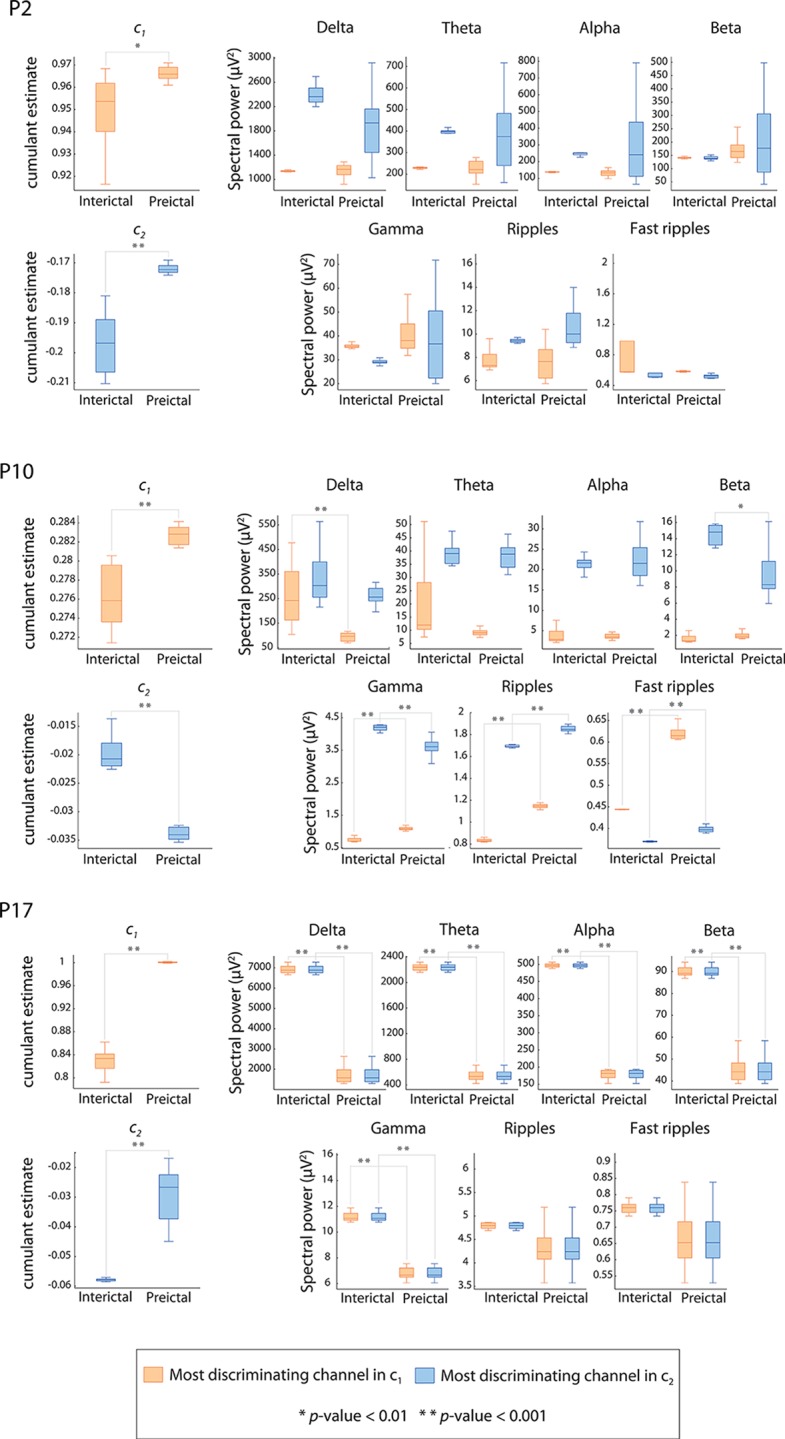
Cumulant vs. spectral power. Box-and-whisker plot (minimum-maximum range) indicating differences in three patients (P2, P10 and P17) between preictal and interictal average 5-min observations of cumulant *c*_1_, cumulant *c*_2_ and the spectral power in the conventional EEG bands. Boxes in orange represent observations from the most discriminating channel in cumulant *c*_1_. Boxes in blue represent observations from the most discriminating channel in cumulant *c*_2_. Significant differences between preictal and interictal observations are denoted by asterisks (* *p*-value < 0.01, ** *p*-value < 0.001).

Using the most discriminating channel in *c*_1_ or in *c*_2_, none of the spectral bands showed a statistically significant difference in every patient. For example, there were no statistically significant difference in the delta power in 8 patients (resp. 10 patients) when there was a statistically significant difference in *c*_*1*_ (resp. *c*_*2*_) using the most discriminating channel in *c*_*1*_ (resp. *c*_*2*_). Fig 4 illustrates the difference in spectral power as compared to that in cumulants in three representative patients (P2, P10 and P17) with respectively the smallest (zero), the median (4) and the highest number (5) of bands showing a significant difference (corrected *p*-value < 0.01).

### Seizure prediction performance using cumulants

**Fig 5:**
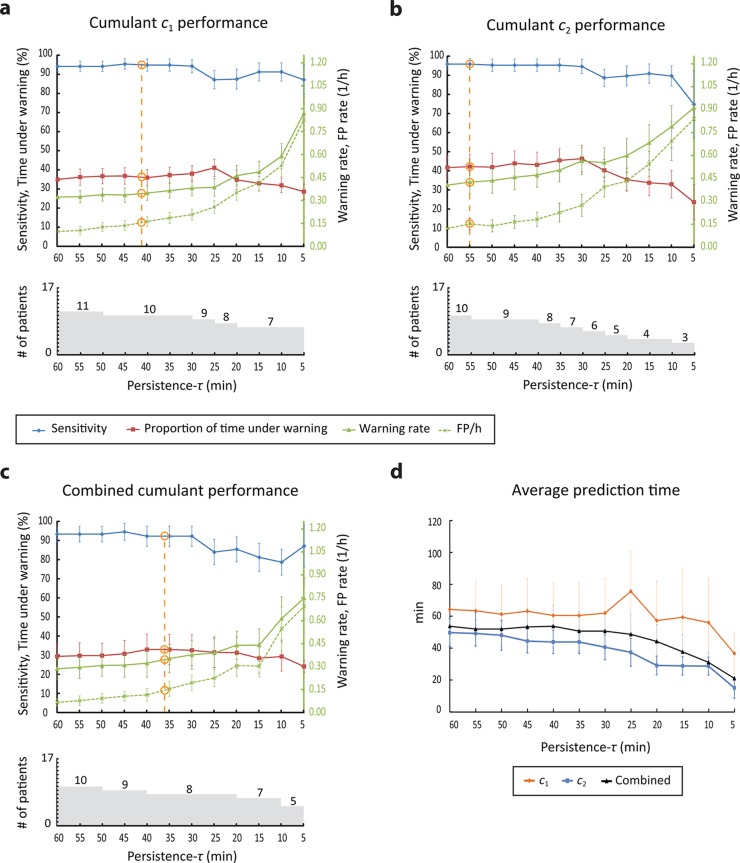
Performance of the seizure prediction method using cumulants and their combination. a-c top: Performance values for the range of persistence-*τ* values analyzed. Orange circles indicate interpolated values of sensitivity, proportion of time under warning and warning rate corresponding to the critical false prediction rate of 0.15/h. a-c bottom: Number of patients in whom seizures are predicted above chance as a function of the persistence-*τ* parameter. d. Average prediction time per patient as a function of the persistence-*τ* parameter.

The performance of the seizure prediction method evaluated on the testing data is shown in Fig 5 for cumulants *c*_1_, *c*_2_ and their combination (respectively feature sets *FS*1, *FS*2 and *FS*3). When cumulant *c*_1_ was used alone, 10 of 17 (59%) patients had their seizures predicted above chance level for persistence-*τ* values 50, 55 and 60 min. Using these persistence-*τ* values, the algorithm achieves an average sensitivity of 94.6%, an average percentage warning time between 34.8 and 36.6%, an average false prediction rate between 0.09 and 0.12/h, and an average warning rate of 0.32/h. The number of patients with predictable (above chance) seizures drops gradually to six when the value of persistence-*τ* is below 15 min. For these persistence-*τ* values, the average sensitivity is 90.3%, the average percentage warning time is 30.1%, the average false prediction rate is 0.59/h, and the average warning rate is 0.65/h.

When cumulant *c*_2_ was used alone, up to eight of 17 (47%) patients had seizures predicted above chance. This is achieved for persistence-*τ* values 55 and 60 min. At these values, the average sensitivity is 96.3% and the average percentage warning time, the average false prediction rate and the average warning rate are respectively 41.9%, 0.13 and 0.44/h. As in the case of cumulant *c*_1_, a gradual decrease in the performance and in the number of patients in whom seizures are predicted above chance is noticed as the value of persistence-*τ* decreases.

The combination of cumulants *c*_1_ and *c*_2_ achieves comparable results to those obtained by either cumulants; seizures are predictable in up to nine of 17 patients (53%). For the set of persistence-*τ* values analyzed, the average sensitivity ranged between 78.3 and 94.1%, the average percentage warning time between 24.4 and 33.1%, the average false prediction rate between 0.07 and 0.69/h, and the average warning rate between 0.29 and 0.75/h.

#### Prediction time

The average prediction time per patient (average across seizures predicted above chance of the time of first preictal change) ranged between 37 and 76 min (median = 61.2 min) across the persistence-*τ* values when cumulant *c*_1_ was used, and between 15.5 and 50.1 min (median = 42.6 min) when cumulant *c*_2_ is used (Fig 5D). With the combination of cumulants, the average prediction time ranged between 21.4 and 54.1 min (median = 51.1 min).

### Seizure prediction performance using combination of cumulants and state similarity measures

**Fig 6:**
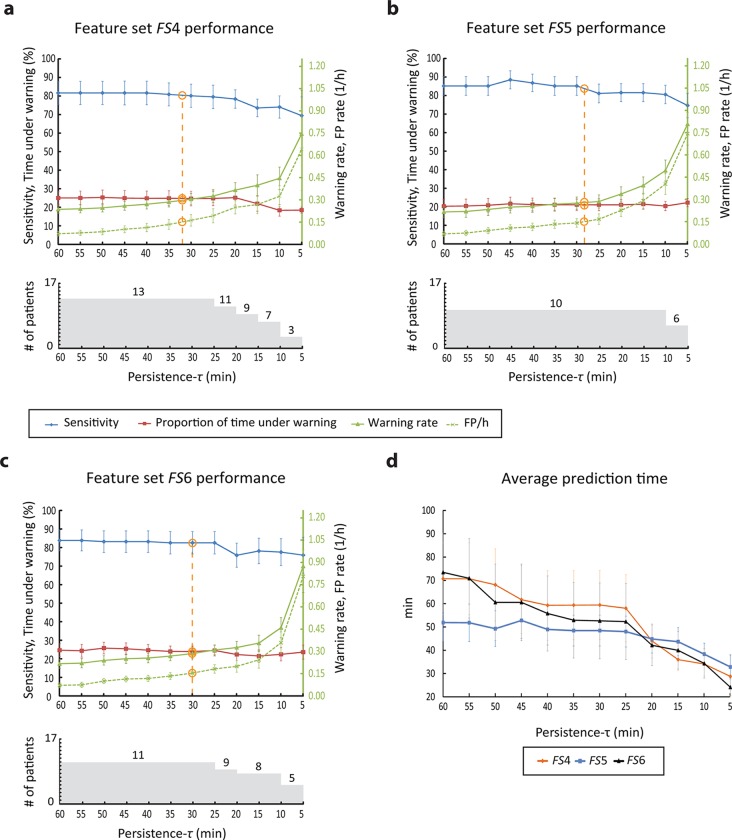
Performance of the seizure prediction method using combination of cumulants and state similarity measures. a-c top: Performance metric values for the range of persistence-*τ* values analyzed when state similarity measures are combined with respectively cumulant *c*_1_ (feature set *FS*4), cumulant *c*_2_ (feature set *FS*5) and both cumulants (feature set *FS*6). Orange circles indicate interpolated values of sensitivity, proportion of time under warning and warning rate corresponding to the critical false prediction rate of 0.15/h. a-c bottom: Number of patients in whom seizures are predicted above chance level as function of the persistence-*τ* parameter, for each of the combination feature sets. d. Average prediction time per patient as a function of the persistence-*τ* parameter for each combination feature set.

Fig 6 shows the performance of the seizure prediction algorithm evaluated on testing data using combinations of cumulants and state similarity measures. The combination of state similarity measures and cumulant *c*_1_ (feature set *FS*4) results in seizures predicted above chance in 13 of 17 patients (76%) for persistence-*τ* values between 25 and 60 min. The average sensitivity is 81%, the average percentage warning time is 25.2%, the average false prediction rate is 0.12/h, and the average warning rate is 0.28/h. The performance and the number of patients with predictable seizures decreases gradually when persistence-*τ* values decreases.

Ten of 17 (59%) patients had seizures predicted above chance using a combination of state similarity measures and cumulant *c*_2_ (feature set *FS*5) for persistence-*τ* values between 10 and 60 min. For these patients, the algorithm achieves an average sensitivity of 84.3%, an average percentage warning time of 21.2%, an average warning rate of 0.3/h, an a false prediction rate between of 0.07 and 0.41/h. When combining state similarity features with both cumulants (feature set *FS*6), seizures were predicted above chance in up to 11 of 17 patients (65%) for the range of persistence-*τ* values between 25 and 60 min. For this range, the average sensitivity is 82.9%, the average percentage warning time is 24.5%, the average false prediction rate is 0.12/h, and the average warning rate is 0.25/h.

#### Prediction time

Fig 6D shows the average prediction time across persistence-*τ* values for feature sets *FS*4, *FS*5 and *FS*6. For all three feature sets, the average prediction time decreases gradually with the value of persistence-τ. Its median value when using *FS*4, *FS*5 and *FS*6 is respectively 59.3, 48.5 and 52.8 min.

### Performance at the critical false positive rate

For a clinically practical seizure prediction method, the false positive rate should be in the order of the patient’s seizure frequency [33,36] which on average is equal to three seizures per day [37]. When medication is discontinued (e.g. during epilepsy monitoring), the average seizure frequency increases to 3.6 seizures per day [38] corresponding to a rate of 0.15 seizures per hour. A false prediction rate above the critical value of 0.15/h in a seizure prediction method is therefore of questionable value. We report in Table 3 the values of the algorithm performance corresponding to the critical false prediction rate. Only performance values corresponding to persistence-*τ* above the values outlined in Table 3 are to be regarded as clinically significant.

**Table 3:** Seizure prediction performance at the critical false prediction rate.

**Feature set**	**# of patients with above-chance predictable seizures**	**Persistence-τ (min)**	**Sensitivity (%)**	**% of time under warning**	**Warning rate (/h)**	**Average prediction time (min)**
*FS*1	9	41.4	95.4	36.1	0.34	61.7
*FS*2	8	55	96.3	42.2	0.43	49.6
*FS*3	7	36.2	91.8	33.1	0.35	51.8
*FS*4	13	33	80.5	25.1	0.3	59.4
*FS*5	10	28.7	84.2	21.3	0.28	43.4
*FS*6	11	30	82.3	23.7	0.28	43.6
*SS* ^1^	7	22.6	81.4	30.5	0.36	50.6

^1^ *SS*: State similarity feature set. These results are reported from previous study.

Values are determined by linear interpolation.

At the critical false positive rate, the best average sensitivity is obtained with feature set *FS*1 (95.4%) for 10 patients, while the best specificity was obtained with feature set *FS*5 (% time under warning = 21.3%) for 10 of 17 patients. Feature *FS*4 achieves the highest number of patients with seizure predicted above chance (13 of 17 patients), with an average sensitivity of 80.5% and an average percentage time under warning of 25.1%. Performance results obtained with feature sets *FS*1 to *FS*6 were overall better than those obtained with state similarity feature in the previous study.

### Surgery outcome and seizure prediction performance

For all feature sets, there was no consistent difference in seizure above-chance predictability between patients who became seizure-free or had rare disabling seizures after surgery (Engel class I and II) and those who continued to have disabling seizures after surgery (Engel class III and IV), in the group of 12 patients who underwent a resective surgery (Fisher’s exact test, p-value > 0.05).

## Discussion

A reliable seizure prediction method with performance levels that would allow for clinical applications remains an unaccomplished task. Ongoing efforts to solve this problem are essentially geared towards finding new descriptors and processing tools of the EEG that would demonstrate a high predictive power. In this context, the current study shows that measures derived from iEEG scaling analysis used together with iEEG state similarity measures can be useful in the prediction of mesial temporal lobe seizures with practical performance levels.

### Summary of findings

Novel descriptors of the intracerebral EEG characterizing the property of scale invariance have been investigated in the prediction of mesial temporal lobe seizures in 17 patients. Cumulants derived from the wavelet leader multifractal analysis of intracerebral EEG showed a statistically significant difference between preictal and interictal epochs in one or more channels in all patients. This difference was not likely attributable to differences in the spectral power suggesting that the cumulants carry a predictive power. However, the association between the difference in cumulants and in the power of some frequency bands in some patients can highlight a possible effect of the state of vigilance on the prediction performance. It is expected that cumulants covary with the spectral power of some but not all frequency bands. This is because cumulants are higher order statistics which embed information about the spectral power (a second order statistic) as well as properties of high statistical order such as long-range dependencies. A correlation analysis between cumulants and the spectral power in the EEG spectral bands should help define the contribution of the spectral power to the predictive power of cumulants. When used as features in our previously proposed seizure prediction method, both cumulants showed prediction capability above chance and with noticeably improved sensitivity (an increase of 17.2% with the first cumulant and 18.3% with the second cumulant, at the critical false positive rate of 0.15/h) over originally used state similarity measures. The number of patients in whom seizures are predicable above chance has also increased from seven to ten (using the first cumulant). Specificity levels were relatively worse using cumulants: a relative increase in the proportion of time under warning of 18.4% using the first cumulant and 38.4% using the second cumulant.

The combination of cumulants slightly outperformed state similarity measures in terms of sensitivity and specificity but did not offer a remarkable improvement over either cumulant used individually. This is probably explained by the relatively worse performance of the second cumulant compared to the first cumulant, suggesting that the predictive information carried by the first cumulant is of higher value and that the multifractality of the iEEG, as captured by the second cumulant, does not change between interictal and preictal states offering less predictive power of seizure occurrence than other properties. Indeed, the combination of the first cumulant and state similarity measures achieved markedly improved prediction performance compared to that achieved with state similarity measures. Particularly, the number of patients in whom seizures are predicted above chance increased from seven to 13 (85.7% increase) when the false prediction rate is fixed at the critical value of 0.15/h. The proportion of time under warning decreased slightly from 30.5 to 25.1%. The sensitivity and the warning rate did not significantly differ.

For seizures predicted above-chance, preictal changes were detected between 15.5 and 76 min on average depending on the feature set and the persistence-*τ* parameter value. Using a combination of state similarity measures and the first cumulant, preictal changes leading to a warning light were detected almost one hour before seizure onset when the false positive rate is fixed at the critical level of 0.15/h. Such a prediction time may not be practical in a seizure advisory system dedicated to warning patients. However, the proposed method should appeal to closed-loop seizure control devices where the intervention time is in the order of the reported prediction time and the levels of required performance are within the range of the achieved performance.

While cumulant *c*_2_ did not show a consistent direction in the difference between preictal and interictal epochs, cumulant *c*_*1*_ was on average significantly higher for the 5-min preictal epochs than for the 5-min interictal epochs in 65% of patients. This preictal increase in the first cumulant indicates that one aspect of the regularity of the iEEG increases before the seizure in these patients. In fact for general cases of scale-invariance models (e.g. multifractal processes), *c*_1_ partially measures the local regularity of a process. Formally, *c*_1_ coincides with the most common value of the local regularity exponent measurable in the process [21]. As *c*_1_ alone might not be sufficient to fully describe the regularity of the iEEG, its preictal increase may not have a visible trace in the iEEG. Such a preictal increase in (one aspect of) the regularity may however point to a more organized and less complex neuronal process underlying the preictal state compared to a less regular and more complex interictal state. Furthermore, this process could presumably emanate from a spatially confined area of the brain such that it is only detectable from few electrode contacts, which could explain why generally only few channels exhibit preictal changes.

Unlike with threshold-based prediction algorithms where the sensitivity and the false prediction rate are positively correlated, classification algorithms using non-linear classification and/or rule-based prediction methods do not necessarily satisfy such correlation. This is the case of the proposed algorithm where a decrease of the sensitivity was associated with an increased false prediction rate leading to a deterioration of performance for a range of seizure prediction horizons. Although a linear discriminant analysis has been used in this study, the rules introduced to decide the final classifications (majority voting decision rule and persistent of alarm lights) are not linear and can lead to uncorrelated sensitivity and specificity results.

### Combination of features is better

Our findings suggest that the preictal change in mesial temporal lobe epilepsy could be better detected if appropriate measures are combined rather than used individually. A better characterization of the preictal state may require different measures that characterize different aspects of the preictal transition. The dynamics of the preictal transition are likely to be complex and accompanied by changes in different properties of the EEG. Quantifying these properties with measures capable of distinguishing the preictal from the interictal state and joining the information carried by each measure should lead to enhanced seizure prediction. This was demonstrated here by combining measures of state similarity and scale invariance properties of the EEG signal. Each set of measures showed individually a predictive power which increased remarkably when both measures were combined.

The level of prediction performance obtained with cumulants is noteworthy, particularly with the first cumulant. To our knowledge, this is the first study to demonstrate a difference in the scale-free dynamics of the intracerebral EEG, as measured by the cumulants, between preictal and interictal epochs and the relevance of the difference in cumulant profiles to seizure prediction. Few studies applied wavelet based cumulants derived from scaling or multifractal analysis to the investigation of the resting state in EEG [39] and in functional magnetic resonance imaging studies [40,41]. Self-similarity, long range dependence and multifractality have been found in magnetoencephalography (MEG) data of healthy patients during rest or task states by applying the WLBMF analysis [42,43]. One study applied wavelet leader cumulants in the classification of EEG patterns in a brain computer interface application [44].

### Scale invariance of the intracerebral EEG

Whether intracerebral EEG reveals scale-free dynamics was not a primary question of this study. We were specifically interested to know if preictal and interictal states manifest different scale-free dynamics as characterized by the cumulants and if any difference could be useful in seizure prediction and its improvement. The profile of the cumulant values obtained for the iEEG data analyzed suggests however that preictal and interictal intracerebral EEG exhibit a scale invariance property. In fact both cumulants *c*_1_ and *c*_2_ had non-zero estimates across all epochs, which indicates that the EEG data could be described by a multifractal process—a scale-free process [21]. Scale invariance is not a characteristic of the epileptic brain. Scale-free activity of the physiological brain has been observed through different modalities [45]. Several studies have suggested that physiological EEG dynamics reflect perpetual brain state transitions, a property known as self-organized-criticality, manifested at the temporal and the spatial scale and following power law distributions [46]. Other studies have shown power law correlations in the EEG alpha wave [47] and long range correlations in the beta wave [48] and scale invariance properties in the functional magnetic resonance imaging signal during resting state [49] and in the EEG during sleep [50]. The observed difference between preictal and interictal cumulant profiles indicates that the transition to seizure is accompanied by a change in the scale-free dynamics of the underlying neuronal mechanisms. Such a change reflects the emergence of a seizure-facilitating state in which the dynamics are characterized by critical time and probably spatial scales. These dynamics might even be observed at the network level where the interaction between units of neurons would show characteristic preictal scale-free dynamics. A multivariate spatiotemporal investigation of cumulant profiles or a fractal connectivity analysis [51–53] would help evaluate this view.

### No correlation with surgery outcome

Predictability and intractability of seizures do not seem to be related as we did not find a correlation between surgery outcome and seizure predictability in the group of patients for whom post-surgery outcome was available. This group was relatively small but our findings are consistent with what has been previously reported [36].

## Conclusion

In summary, we improved the performance of a previously proposed seizure prediction method by using a combination of features. New descriptors of intracerebral EEG ― the wavelet leader cumulants based on a novel scaling analysis approach ― were efficiently employed to improve seizure prediction performance. The combination of state similarity measures and cumulants led to seizures predicted above chance in an additional six of 17 patients compared to previous study, with enhanced specificity and maintained sensitivity levels. These results suggest that seizures of many patients with mesial temporal lobe epilepsy can be predicted with clinically practical performance and encourage prospective validation on a larger cohort of patients. Given the relatively long prediction horizons evaluated, the improved method can be particularity useful for responsive seizure control devices based on seizure prediction.

## Supporting Information

S1 Appendix Mathematical formalism of the wavelet leader based scaling analysis. (DOCX)

S1 Fig Illustration of the wavelet leader definition. The wavelet Leader *L*_*X*_ (red dot) is the supremum of the discrete wavelet coefficients *d*_*X*_ (grey dots) in the time neighborhood 3*λ*_*j*,*k*_ over all finer scales 2^*j’*^ < 2^*j*^ (area in grey). (adapted from [30]).
(EPS)

S1 Table Training and testing EEG data. (XLSX)
