# Supplementary material for: Scale Invariance Properties of Intracerebral EEG Improve Seizure Prediction in Mesial Temporal Lobe Epilepsy
Source: PLoS One. 2015 Apr 13;10(4):e0121182. doi: 10.1371/journal.pone.0121182 (PMC4395084; doi:10.1371/journal.pone.0121182)
Supplement: S1 Appendix — (DOCX) [file pone.0121182.s001.docx]

# **Appendix. Mathematical formalism of the wavelet leader based scaling analysis**

## **Wavelet leaders**

Assuming a discrete process *X(t)* and a mother wavelet function *ψ_0_(t)* with a compact time support, the coefficients of the discrete wavelet transform (DWT) of *X*, *d_X_(j,k)* [54] are given by:

(1)

Let *λ = λ _j,k_ =*[*k2^j^,* (*k+1*)*2^j^*) be a dyadic intervals of indices and $3\lambda=\lambda_{j,k-1}\cup\lambda_{j,k}\cup\lambda_{j,k+1}$ the union of the interval *λ* with its adjacent dyadic intervals. The wavelet leaders [55] are the quantities *L_X_(j,k)* defined from the discrete wavelet coefficients as:

$L_{X}\left(j,k\right)=sup_{\lambda'\subset3\lambda}\left|d_{X,\lambda'}\right|$ (2)

*L_X_(j,k)* is the largest discrete wavelet coefficient computed over all finer scales 2*^j’^* ≤ 2*^j^* in the time neighborhood 3*λ* (S1 Fig.).

## **Log-cumulants**

For a given analysis scale, the time averages (across the *n_j_* samples of *X(t)* at the scale 2*^j^*) of the *q*^th^ power of the wavelet leaders *L_X_(j,k)* are referred to as the structure functions:

$S^{L}\left(j,q\right)=\frac{1}{n_{j}}\sum_{k=1}^{n_{j}} L_{X}\left(j,k\right)^{q}$ (3)

Under mild uniform Hölder regularity condition on *X*(*t*), it has been demonstrated [55] that the structure functions are related to the scaling exponents $\zeta\left(q\right)$ by a power law in the limit 2*^j^* → 0:

$S^{L}\left(j,q\right)=F_{q}2^{j\zeta\left(q\right)\text{ }}$ (4)

The structure functions *S^L^*(*j*,*q*) could be expressed as the sample mean estimators for the ensemble averages . Using the second characteristic functions of the distributions of random variables ln *L_X_*(*j*,∙), eq. (4) could be rewritten as:

(5)

where $C_{p}^{j}$ are the cumulants of order p ≥ 1 of ln *L_X_*(*j*,∙).

Eq. (5) implies that:

$\forall p \geq1:C_{p}^{j}=c_{p}^{0}+c_{p}ln2^{j}$ (6)

Replacing $C_{p}^{j}$ in eq. (5) by their expression in eq. (6) yields:

$\zeta\left(q\right)=\sum_{p=1}^{\infty} c_{p}\frac{q^{p}}{p!}\text{ }$ (7)

$c_{p}$ is denoted the log-cumulant (or simply the cumulant) of order *p*.
